# Physically and Chemically Crosslinked, Tannic Acid Embedded Linear PEI-Based Hydrogels and Cryogels with Natural Antibacterial and Antioxidant Properties

**DOI:** 10.3390/biomedicines11030706

**Published:** 2023-02-25

**Authors:** Mehtap Sahiner, Aynur Sanem Yilmaz, Sahin Demirci, Nurettin Sahiner

**Affiliations:** 1 Department of Bioengineering, Faculty of Engineering, Canakkale Onsekiz Mart University Terzioglu Campus, Canakkale 17100, Turkey; 2 Department of Chemistry, Faculty of Science, Nanoscience and Technology Research and Application Center, Canakkale Onsekiz Mart University Terzioglu Campus, Canakkale 17100, Turkey; 3 Department of Chemical and Biomedical Engineering, Materials Science and Engineering Program, University of South Florida, Tampa, FL 33620, USA; 4 Department of Ophthalmology, Morsani College of Medicine, University of South Florida, 12901 Bruce B Downs B. Downs Blv., MDC 21, Tampa, FL 33612, USA

**Keywords:** linear polyethyleneimine, polyethyleneimine/tannic acid hybrid, antioxidant, antibacterial, tannic acid release, controllable drug release, hydrogel/cryogel

## Abstract

Linear polyethyleneimine (L-PEI) was obtained from the acidic hydrolysis of poly(2-ethyl-2-oxazoline) and employed in the synthesis of physically crosslinked L-PEI hydrogel, PC-L-PEI^H^, chemically crosslinked L-PEI hydrogel, CC-L-PEI^H^, and cryogels, CC-L-PEI^C^. The preparation of L-PEI-based hydrogel networks was carried out in two ways: 1) by cooling the L-PEI solution from 90 °C to room temperature, and 2) by crosslinking L-PEI chains with a crosslinker, glycerol diglycidyl ether = 20 °C for CC-L-PEI^C^. Furthermore, a polyphenolic compound, tannic acid (TA), with superior antibacterial, antioxidant, and anti-inflammatory properties as an active biomedical functional agent, was encapsulated during the synthesis process within L-PEI-based hydrogels and cryogels, at 10% and 25% (w/w) based on the L-PEI amount. A linear and higher TA release was observed from physically crosslinked PEI-based hydrogels containing 10% and 25% TA-containing PC-L-PEI/TA^H^ within 6 h, with 9.5 ± 05 mg/g and 60.2 ± 3.8 mg/g cumulative released amounts, respectively. A higher antioxidant activity was observed for 25% TA containing PC-L-PEI/TA^H^ with 53.6 ± 5.3 µg/mL total phenol content and 0.48 ± 0.01 µmole Trolox equivalent/g. The minimum bactericidal concentration (MBC) of PC-L-PEI^H^ and CC-L-PEI^C^ networks against both *E. coli* (ATCC 8739) and Gram-positive *B. subtilis* (ATCC 6633) bacteria was determined at 5 mg/mL, whereas the MBC value of 10 mg/mL for CC-L-PEI^H^ networks against the same bacteria was achieved.

## 1. Introduction

Cationic polymers have emerged as significant structures with their use as one of the go-to materials for environmental applications, where they may be utilized to electrostatically separate negatively charged target components from industrial wastes [1,2,3]. Accordingly, cationic polymers afford many active groups such as amines, sulfonium, and phosphonium and more adaptable chain architectures than traditional inorganic cations [4]. Additionally, by properly designing the initial monomeric precursors as well as employing the post-modification technique, it is possible to manage cationic polymers’ functional qualities, such as molecular weight, charge distribution, biocompatibility, and so on [5,6,7]. Polyethyleneimine (PEI), one of the most attractive polycations, is well-recognized for its versatility and highly positively charged nature in a variety of formulations. Previous research has demonstrated that PEI is capable of interacting with metal ions and negative ions through complexing or neutralization [8]. Furthermore, PEI is compatible with a variety of substrates and may be applied as functional coatings [9]. Additionally, PEI has abundant surface amine groups in a linear or branching structure and is commonly employed in biomedical imaging, gene therapy, as an antibacterial agent, and in controlled drug release systems [10,11,12,13]. For diverse biological applications, PEI has been employed to generate a variety of organic/organic or organic/inorganic hybrid materials [14,15,16,17].

Tannic acid (TA) is a hydrolyzable amphiphilic tannin found in various natural sources such as gallnuts, grapes, tea, and coffee [18]. TA is an FDA-approved polyphenolic compound and has been safely used as a food additive, bio-sorbent, and animal feed due to its excellent properties such as metal chelation, antioxidant activity, and polymerization [18,19]. A wide variety of therapeutic effects of TA have been reported, including radical scavenging, anti-inflammatory, neuro-protective, and antiviral effects [20]. Moreover, TA has been proven to target specific cancer cell lines in neoplastic diseases [21]. TA has high inhibitory effects against certain microorganisms such as yeast, fungi [22], Gram-positive, and Gram-negative bacteria [19]. These favorable features make TA a promising pharmaceutical candidate in various biomedical fields [19]. However, clinical applications of TA have been limited due to its low lipid solubility, low bioavailability, and short duration. After ingestion, it has poor bioavailability due to its large size, high affinity to bind plasma proteins, and low lipid solubility [23]. Thanks to its intrinsic structure composed of hydroxyl and carboxyl groups, TA could interact with organic, inorganic, natural, or synthetic compounds and hydrophilic and hydrophobic materials via cross-linking [18,24].

Novel drug delivery systems have been designed to deliver TA to improve its biomedical and pharmaceutical applications [21]. For example, TA was combined with anticancer drug molecules to form nanoparticles [25,26]. These nanocomplexes improved the anticancer activity, showed good cytotoxicity, reduced IC50 values, and increased cellular uptake of the drugs [24,25,26]. Tannic acid was proven to preserve its high antioxidant activity when encapsulated in nanoparticles and exhibited a progressive in vitro release profile as desired [27]. Previously published studies supported the idea that controllable release of TA from composites/matrices, and thus sustainable antimicrobial and antioxidant activities, could be achievable [28].

Here, linear polyethyleneimine (L-PEI) was synthesized via the acidic hydrolysis of poly(2-ethyl-2-oxazoline) (PEOX) under reflux. Then, the prepared L-PEI was used in the preparation of physically crosslinked L-PEI hydrogel (PC-L-PEI^H^), chemically crosslinked L-PEI hydrogel (CC-L-PEI^H^), and in a cryogel form (CC-L-PEI^C^) using glycerol diglycidyl ether (GDE) as the crosslinker. Moreover, physically and chemically cross-linked L-PEI/TA hydrogels and cryogels containing 10% and 25% w/w tannic acid (TA) were also prepared to demonstrate TA release from the prepared networks in a preferred amount and time course. The comparison of TA release from physically and chemically crosslinked L-PEI/TA hydrogels and cryogels was done in pH 7.4 phosphate buffer solution (PBS) at 37 °C. Also, the antioxidant properties of L-PEI/TA hydrogels and cryogels were investigated via total phenol content (TPC) and ABTS^+^ scavenging assays. Antibacterial properties of physically and chemically crosslinked L-PEI and L-PEI/TA hydrogels and cryogels against Gram-negative *Escherichia coli* (*E. coli*, ATCC 8739) and Gram-positive *Bacillus subtilis* (*B. subtilis*, ATCC 6633) bacteria were also tested employing macro-dilution and disc diffusion techniques. The results of this study could serve as primary results for in vivo experiments.

## 2. Materials and Methods

### 2.1. Materials

Poly(2-ethyl-2-oxazoline) (PEOX, Mn:50000, PDI 3-4, Sigma Aldrich, St. Louis, MO, USA), hydrochloric acid (HCl, 37%, Carlo Erba, Val-de-Reuil, France), and sodium hydroxide (NaOH, 99%, AFG Bioscience, Northbrook, IL, USA) were used for the synthesis of linear polyethyleneimine (L-PEI). Glycerol diglycidyl ether (GDE; Technical grade; Sigma Aldrich, Tokyo, Japan) was used as a crosslinker for the preparation of chemically crosslinked L-PEI-based hydrogels and cryogels. Tannic acid (TA, 99%, Sigma Aldrich, Bornem, Belgium) was used as an active drug ingredient. In the antioxidant activity tests, Folin-Ciocalteau phenol reagent (FC, 2N, Sigma-Aldrich, Buchs, Switzerland) and sodium bicarbonate (NaHCO_3_, 99%, Sigma-Aldrich, Saint Louis, MO, USA) were used for the TPC test, and 2,20-Azino-bis(3-ethylbenzothiazoline-6-sulfonic acid) diammonium salt (ABTS^+^, 98%, Sigma-Aldrich, Markham, ON, Canada) and potassium persulfate (KPS, 99%, Carlo Erba, Val-de-Reuil, France) were used for the TEAC test. For the antimicrobial activity tests, Gram-negative bacteria *Escherichia coli* (*E. coli*, ATCC 8739) and Gram-positive bacteria *Bacillus subtilis* (*B. subtilis*, ATCC 6633) were obtained from the Microbiology Department of the School of Medicine at Canakkale Onsekiz Mart University. As bacterial growth medium, nutrient agar (NA, Condolab, Madrid, Spain), and nutrient broth (NB, Merck, Darmstadt, Germany) were used as received.

### 2.2. Synthesis of L-PEI

The synthesis of L-PEI was carried out via hydrolysis of PEOX under acidic conditions by following the literature [29]. In brief, 10 g of PEOX were weighed and added to a 250-mL round-bottomed flask containing 50 mL of water. After that, 50 mL of a 36% concentrated HCl solution was placed on the prepared solution of PEOX in 50 mL of water and stirred at room temperature for 10 min. The flask was then placed in a previously heated magnetic stirrer to 100 °C with a reflux setup and stirred at 800 rpm for 14 h. Finally, the obtained blurred solution was placed into 400 mL of ice-cold water and stirred until it became a transparent solution. Then, the ice-cold 4 M NaOH solution was dropwise added to this solution. The obtained precipitation has a pH range of 10–11, confirming the essential nature of L-PEI chains. The L-PEI was precipitated with centrifugation at 10,000 rpm and 4 °C for 10 min. The precipitated L-PEI was washed with water two times. Lastly, the obtained L-PEI was dried in a freeze-dryer for two days and stored in closed tubes for further usage.

### 2.3. Synthesis of L-PEI-Based Hydrogels and Cryogels

#### 2.3.1. Synthesis of Physically Crosslinked L-PEI and L-PEI/TA Hydrogels

The synthesis of physically crosslinked L-PEI, PC-L-PEI^H^, and TA-containing L-PEI/TA (PC-L-PEI/TA^H^) hydrogels was carried out according to the literature with some modifications [29]. Firstly, 1 g of L-PEI was dissolved in 7.5 mL of water at 90 °C under continuous stirring at 500 rpm, and 2.5 mL of a 1 M NaOH solution was added to the reaction medium. On the other hand, the prepared TA solutions, at 10% and 25% w/w according to the weight of L-PEI, in 2.5 mL of 1 M NaOH, were added drop-by-drop to the L-PEI solutions (7.5 mL in water) at 90 °C under continuous stirring at 500 rpm and stirred for 5 min more at 90 °C. Finally, the prepared L-PEI and L-PEI/TA solutions were placed into plastic straws (7 mm in diameter) and cooled to room temperature. Finally, the prepared PC-L-PEI^H^ and PC-L-PEI/TA^H^ were cut into similar shapes and sizes and dried in a freeze-dryer. Before the drying process, all PC-L-PEI-based hydrogels were swilled out once to remove unreacted reactants. The prepared PC-L-PEI/TA^H^ containing 10 and 25% w/w TA were named PC-L-PEI/TA^H^-1 and PC-L-PEI/TA^H^-2, respectively.

#### 2.3.2. Synthesis of Chemically Crosslinked L-PEI, L-PEI/TA Hydrogels, and Cryogels

For the synthesis of chemically crosslinked L-PEI (CC-L-PEI^H^) hydrogels, the prepared L-PEI solutions at a concentration of 0.1 g/mL in 10 mL of 0.25 M NaOH were dissolved at 90 °C and stirred at 500 rpm. Then, 0.237 mL of GDE (5% mol ratio relative to the L-PEI repeating unit (-CH_2_CH_2_NH-, 43 g/mol) was added to the reaction medium after observing the clear L-PEI solution, and after vortexing for 30 s, it was quickly placed on plastic pipettes of 7 mm diameter. The plastic pipettes were kept at room temperature for 24 h for the completion of crosslinking reactions.

The same method was used for the synthesis of chemically crosslinked L-PEI (CC-L-PEI^C^) cryogels. Plastic pipettes were placed into a freezer at −20 °C and kept there for 24 h to complete crosslinking reactions.

Moreover, the chemically crosslinked L-PEI/TA hydrogel (CC-L-PEI/TA^H^) and cryogel (CC-L-PEI/TA^C^) were also prepared as mentioned above. For this purpose, 1 g of L-PEI was dissolved in 7.5 mL of water at 90 °C, and the prepared TA solutions in 2.5 mL of 1 M NaOH solutions (TA contents are 10 and 25% w/w with respect to the weight of L-PEI) were added dropwise to the L-PEI solutions and stirred for 5 more minutes at 90 °C. Next, 0.237 mL of GDE (5% mol ratio relative to the L-PEI repeating unit) was added to the reaction medium, and after vortexing for 30 s, it was quickly placed on plastic pipettes (7 mm in diameter). The plastic pipettes were kept at room temperature for 24 h for the formation of CC-L-PEI/TA^H^ and placed into the freezer for the preparation of CC-L-PEI/TA^C^, separately.

Finally, the chemically crosslinked L-PEI and L-PEI/TA hydrogels and cryogels were cut into similar shapes and sizes and dried in the freeze dryer. Before the drying process, all chemically crosslinked hydrogels and cryogels were swilled out once to remove unreacted reactants. The prepared CC-L-PEI/TA^H^ and CC-L-PEI^C^, containing 10% and 25% w/w TA, were denoted as “−1” and “−2” respectively, as mentioned in physically crosslinked hydrogels.

### 2.4. Characterization

A fourier transform infrared radiation (FT-IR, Nicolet iS10, Thermo, Waltham, MA, USA) spectrometer was used for the assessment of the functional group of all L-PEI hydrogels and cryogels via the attenuated total reflector (ATR) technique in the wavenumber range of 4000–650 cm^−1^.

A thermogravimetric analyzer (TGA, SII TG/DTA 6300, Seiko, Tokyo, Japan) was used for the comparison of the thermal stabilities of the prepared L-PEI-based hydrogels and cryogels. For this purpose, a certain amount of sample (3–5 mg) was placed into a ceramic TGA cuvette and heated up to 100 °C to remove water or moisture from the structure under a 20 mL/min N_2_ gas flow. After that, the samples were heated up to 700 °C with a 10 °C/min heating rate under a 20 mL/min N_2_ gas flow.

Swelling % (S%), porosity % (P%), pore volume % (PV%), and gel yield % values of the prepared L-PEI-based hydrogels and cryogels were calculated with the following equations.

S% = [(W_s_ − W_d_)/W_d_] × 100 (1)

P% = [(W_s_ − W_sq_)/W_s_] × 100 (2)

VP% = (W_ch_ − W_d_)/(W_d_ × d_ch_) × 100 (3)

Gel Yield% = (W_product_/W_reactant_) × 100 (4)

where “W_s_” is the weight of swollen, “W_d_” is the weight of dry, “W_sq_” is the weight of squeezed forms of L-PEI-based hydrogels and cryogels, and “W_ch_” is the weight of L-PEI-based hydrogels and cryogels in cyclohexane. In addition, “W_product_” is the weight of synthesized L-PEI-based hydrogels and cryogels, whereas “W_reactant_” is the weight of precursors in the preparation of L-PEI-based hydrogels and cryogels.

### 2.5. TA Release from L-PEI/TA Hydrogels and Cryogels

The release of TA from the prepared L-PEI/TA hydrogels and cryogels was carried out in a PBS solution at pH 7.4 and 37 °C. For this purpose, certain amounts of 10% and 25% w/w TA containing L-PEI/TA hydrogels and cryogels were placed into dialysis membranes with 2 mL of PBS, and dialysis membranes were placed into erlenmeyers containing 98 mL of pH 7.4 PBS, separately. After that, the erlenmeyers were placed into a shaking bath at 37 °C, and the released TA amount was determined from the calibration curve of TA in pH 7.4 PBS at 275 nm wavelength at various time intervals via a UV-Vis spectrophotometer (SP-UV300SRB, Spectrum, Quanzhou, China). The measurements were done in triplicate, and the results were given as average values with standard deviations.

### 2.6. Antioxidant Properties of L-PEI-Based Hydrogels and Cryogels

To investigate the antioxidant properties of tannic acid containing L-PEI-based materials, they were separately put into fresh 20 mL of PBS solution in tubes and kept at 37 °C for 3 h. Then, the supernatant solutions were taken from the tubes and read by a UV-Vis spectrophotometer at 275 nm to determine the released amount of tannic acid content.

#### 2.6.1. Total Phenol Content Assay

The total phenolic contents of the prepared materials were measured using the Folin–Ciocalteu (FC) method described by Kowalczyk et al. [30]. First, 100 µL of samples at certain concentrations (obtained after a 3-h release time) were reacted with 1.25 mL of a 0.2 N solution of FC reagent for 4 min. After this, 1 mL of a 0.7 M sodium bicarbonate solution was added to this mixture and kept in the dark condition for 2 h. Then, the total phenol content of the prepared materials was measured using a UV-Vis spectrophotometer (T80^+^ PG Instrument) at 760 nm. The antioxidant activity results of the samples were expressed by using a calibration curve of gallic acid as µg/mL gallic acid equivalent. The analysis was performed twice, and the values were given as the mean values.

#### 2.6.2. ABTS^+^ Scavenging Assay

The antioxidant capacities of prepared materials were also measured by ABTS**^+^** scavenging assay in accordance with the literature [30]. An ABTS^+^ radical solution was prepared by mixing 3 mL of 2.45 mM potassium persulfate aqueous solution with 9 mL of 7 mM ABTS^+^ aqueous solution, and the mixture was kept in the dark for 16 h in a cool place. The stock of ABTS^+^ was diluted with PBS to obtain an absorbance value of 0.7 ± 0.05 at 734 nm. After this, 3000 µL of diluted ABTS^+^ solution was reacted with various amounts of these suspensions (50–400 µL) for 6 min. At the end of 6 min, the values with a 20−80% reduction of the blank absorbance at 734 nm were determined. Trolox equivalent antioxidant capacity (TEAC) values were determined against the Trolox standard curve and defined as “µmol Trolox equivalent/g.” The test was repeated three times, and the values were given as averages with standard deviations.

### 2.7. Antimicrobial Activity Tests of L-PEI-Based Hydrogels and Cryogels

The antimicrobial properties of L-PEI-based hydrogels and cryogels were determined by the macro-dilution method and a zone of inhibition experiment against *E. coli* (ATCC 8739) and *B. subtilis* (ATCC 6633) as Gram-negative and Gram-positive bacteria, respectively, by following the previous procedures [31,32]. Reisner and Woods discovered that only seven microorganisms cause 85–90% of all clinically reported illnesses, with *E. coli* being responsible for almost half of all infections [33]. Also, *B. subtilis* bacteria in patients with compromised immune status have been reported to cause infections such as bacteremia, endocarditis, pneumonia, and septicemia [34]. Prior to testing, L-PEI-based hydrogels and cryogels were sterilized for 10 min under the UV light (λ = 420 nm).

#### 2.7.1. Macro-Dilution Assay

Fresh 24 h cultures of *E. coli* and *B. subtilis* strains were suspended in 5 mL of nutrient broth (NB) liquid growth medium and mixed with a mini-shaker to adjust to 0.5 McFarland’s standard containing 10^8^ CFU mL^−1^ (colonies forming units). Then, 50, 100, and 200 mg of sterilized L-PEI-based hydrogel and cryogel samples were weighed and placed into 10 mL of NB in tubes to obtain 5, 10, and 20 mg/mL concentration samples, respectively. After this step, 100 µL of bacterial suspensions were added into tubes and incubated at 35 °C for 18–24 h. After the incubation, the broth media in the tubes were diluted with 0.9% physiological saline solution to count the colony numbers. Then, 100 µL of diluted broth media containing bacteria were seeded onto nutrient agar solid growth medium and kept at 35 °C in the incubator for 24 h. Finally, the bacterial growth was evaluated by counting the colonies.

#### 2.7.2. Zone of Inhibition Experiments

To determine the zone of inhibition of the prepared L-PEI-based hydrogel and cryogel samples, 100 µL of *E. coli* and *B. subtilis* suspensions containing approximately 10^8^ CFU ml^−1^ were poured onto nutrient agar solid growth medium in 90 mm petri dishes and spread. Immediately, sterilized L-PEI-based hydrogel and cryogel samples weighing exactly 1.5 mg were gently placed onto nutrient agar, and the petri dishes were incubated at 35 °C for 24 h. After the incubation, samples were carefully removed from the plates, and zone diameters of inhibition were measured. Antibacterial activity assays were repeated twice on plates.

## 3. Results and Discussion

### 3.1. Synthesis and Characterization of L-PEI-Based Hydrogels and Cryogels

**Figure 1:**
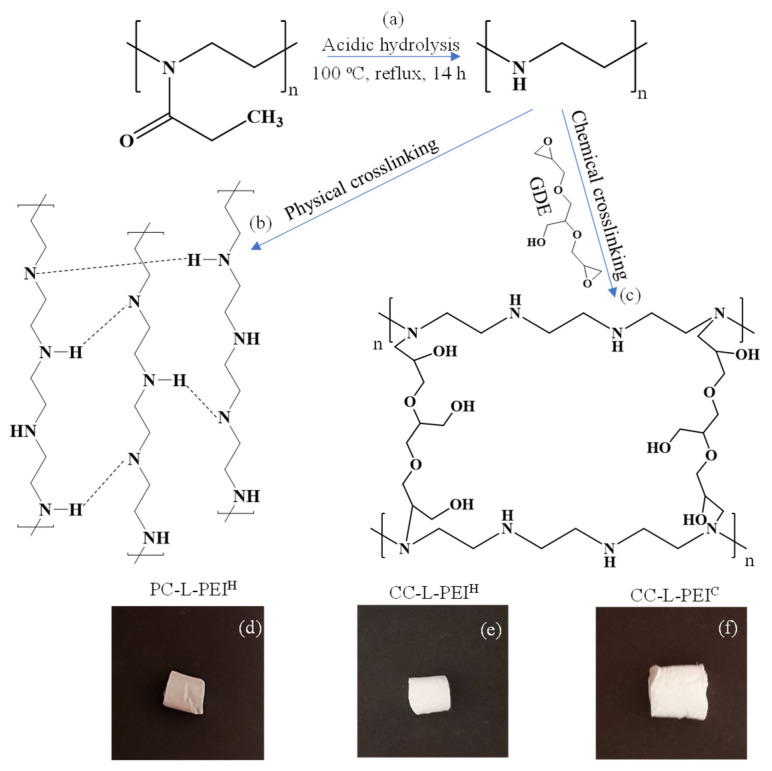
The schematic presentation of the synthesis of (**a**) L-PEI chains, (**b**) PC-L-PEI^H^, and (**c**) CC-L-PEI^H^/CC-L-PEI^C^, and digital camera images of (**d**) PC-L-PEI^H^, (**e**) CC-L-PEI^H^, and (**f**) CC-L-PEI^C^.

For the synthesis of linear polyethyleneimine (L-PEI)-based hydrogels and cryogels, L-PEI was synthesized following the method reported by Soradech et al. [29]. For this purpose, L-PEI was obtained from the hydrolysis of PEOX in acidic conditions at 100 °C. The possible reaction was illustrated in Figure 1a. The removal of the propionaldehyde group from the polymer chains of PEOX in the presence of HCl and the hydrolysis of nitrogen provide for the formation of L-PEI chains [35,36]. The prepared L-PEI chains were used as a precursor to synthesize L-PEI-based hydrogels and cryogels. Earlier, Soradech et al. synthesized physically crosslinked PEI cryogels via freeze-thaw technique with intramolecular NH^…^N hydrogen bonds [29].

In this paper, three types of L-PEI-based materials were synthesized: physically crosslinked L-PEI hydrogels (PC-L-PEI^H^), chemically crosslinked L-PEI hydrogels (CC-L-PEI^H^), and chemically crosslinked L-PEI cryogels (CC-L-PEI^C^). The schematic presentation of the preparation of L-PEI-based hydrogels and cryogels is illustrated in Figure 1b,c respectively. The prepared PC-L-PEI^H^ was physically crosslinked with NH^…^N hydrogen bonding, as reported in the literature [29]. On the other hand, the chemically crosslinked CC-L-PEI^H^ and CC-L-PEI^C^ structures were crosslinked with GDE via ring-opening reactions, as reported in the literature [8]. The amine groups of L-PEI chains reacted with the epoxy groups of GDE, and CC-L-PEI^H^ and CC-L-PEI^C^ structures were prepared at room temperature and −20 °C, respectively. The digital camera images of PC-L-PEI^H^, CC-L-PEI^H^, and CC-L-PEI^C^ structures are also given in Figure 1d–f respectively. Cryogels are a special type of common hydrogel with interconnected, super porous structures, higher flexibility, elasticity, and mechanical strength, and fast response capabilities to environmental stimuli [37]. Therefore, cryogels are commonly used in tissue engineering, biomolecule separation, enzyme immobilization, etc. [38].

**Figure 2:**
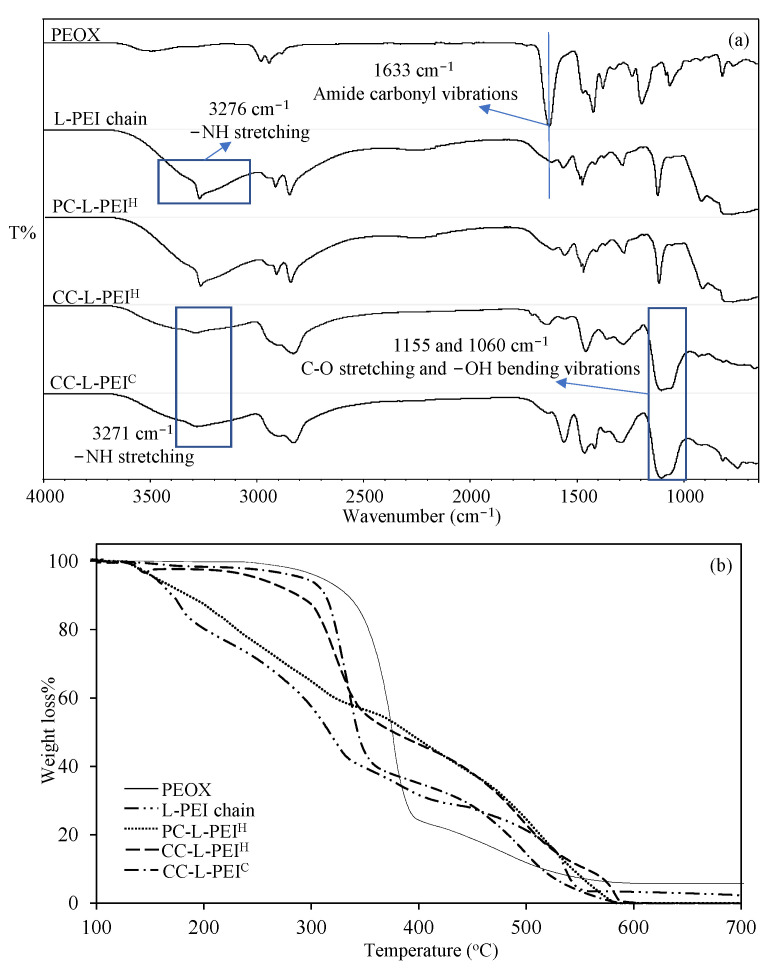
The comparison of (**a**) the FT−IR spectrum and (**b**) the TGA thermograms of PEOX, L-PEI chains, and L-PEI-based hydrogels and cryogels.

To confirm the synthesis of L-PEI from PEOX and the preparation of PC-L-PEI^H^, CC-L-PEI^H^, and CC-L-PEI^C^ structures, FT-IR spectra are given in Figure 2a. It was clearly seen that the most distinct peak was the amide carbonyl vibration at 1633 cm^−1^ on the FT−IR spectrum of PEOX. Also, the disappearance of this peak after hydrolysis in acidic media and the observed peak at 3276 cm^−1^ which is attributed to NH stretching vibration, show the successful conversion of PEOX to L-PEI [35]. The FT−IR spectrum of PC-L-PEI^H^ structures overlapped exactly with the FT-IR spectrum of L-PEI chains, as expected. There was also an NH stretching vibration peak at 3271 cm−^1^ that was clearly seen in CC-L-PEI^H^ and CC-L-PEI^C^ structures. There were also two new peaks observed at 1155 and 1060 cm^−1^ which are assigned to C−O stretch and −OH bending vibration as a result of crosslinking with GDE [8].

The comparisons of the thermal behaviors of PEOX, L-PEI chains, and L-PEI-based hydrogels and cryogels are given in Figure 2b. It was seen that the PEOX started to degrade around 320 °C, and between 320–380 °C a 75% weight loss was observed. The degradation of PEOX continued slowly between 385–550 °C with a 92% cumulative weight loss. On the other hand, the L-PEI chains started to degrade between 150–190 °C with 13% weight loss and continued to degrade between 240–330 °C with 54% and 340–590 °C with 92% cumulative weight losses, respectively. It was also observed that the prepared PC-L-PEI^H^ started to degrade at 150 °C, with a weight loss of 42% at 330 °C and a second degradation step between 360–570 °C, with a cumulative weight loss of more than 99%. On the other hand, the chemically crosslinked CC-L-PEI^H^ and CC-L-PEI^C^ were almost thermally stable up to 230 °C and 280 °C, respectively, and showed similar degradation profiles, which were two-step degradations between 240–350 and 290–350 °C with 63% and 57% weight losses, respectively, and 460–590 °C and 450–580 °C with >99% cumulative weight losses, respectively. It can be concluded from TGA thermograms of PEOX, L-PEI chains, and L-PEI-based hydrogels and cryogels that the chemical crosslinking of L-PEI chains to produce CC-L-PEI^H^ and CC-L-PEI^C^ increased the thermal stability of L-PEI chains with almost no degradation at more than 200 °C levels.

Moreover, the S%, P%, PV%, and gel yield % values for PC-L-PEI^H^, CC-L-PEI^H^, and CC-L-PEI^C^ networks were also calculated and summarized in Table 1. The PC-L-PEI^H^, CC-L-PEI^H^, and CC-L-PEI^C^ networks swelled in 1 h, 3 h, and 1 min, respectively. The S% values for PC-L-PEI^H^, CC-L-PEI^H^, and CC-L-PEI^C^ networks were also calculated as 512 ± 54, 976 ± 107, and 1154 ± 142%, respectively.

The P% values for PC-L-PEI^H^, CC-L-PEI^H^, and CC-L-PEI^C^ networks were calculated as 51 ± 4, 19 ± 2, and 57 ± 2%, whereas the PV% values were calculated as 34 ± 3, 7 ± 1, and 57 ± 2%, respectively. Moreover, the gel yield % of all L-PEI-based networks was calculated to be more than 95%. It is apparent that the chemically crosslinked hydrogels and cryogels have a higher swelling degree than the physically crosslinked hydrogels. On the other hand, the P% value of chemical crosslinked hydrogel (CC-L-PEI^H^) was less than PC-L-PEI^H^ hydrogel and as expected CC-L-PEI^C^ cryogel had the highest P% value, 63 ± 5. The PV% values were also similar to the P% values. Furthermore, the gel yield % values of the prepared hydrogel materials were high and calculated at about 96%.

### 3.2. Synthesis and Characterization of L-PEI/TA-Based Hydrogels and Cryogels

**Figure 3:**
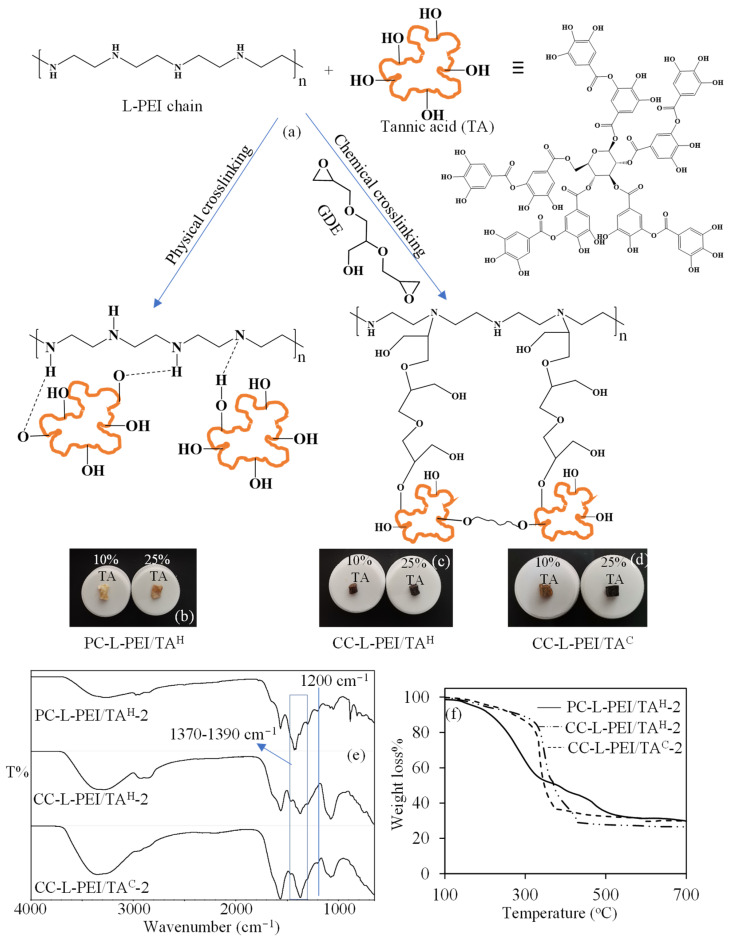
(**a**) the schematic presentation of the synthesis of physically and chemically crosslinked L-PEI/TA networks, digital camera images of 10 and 25% TA containing (**b**) PC-L-PEI/TA^H^, (**c**) CC-L-PEI/TA^H^, and (**d**) CC-L-PEI/TA^C^, and comparisons of (**e**) the FT–IR spectrum, and (**f**) TGA thermograms of L-PEI/TA networks.

The synthesis of TA-containing L-PEI-based hydrogels and cryogels was also prepared as mentioned above. The TA solutions, at 10 and 25% w/w in 2.5 mL of 1 M NaOH solution (based on L-PEI), were added to L-PEI solutions, and both physically and chemically crosslinked L-PEI/TA hydrogels and cryogels were synthesized. The schematic presentation of the preparation of PC-L-PEI/TA^H^, CC-L-PEI/TA^H^, and CC-L-PEI/TA^C^ is illustrated in Figure 3a. For the preparation of PC-L-PEI/TA^H^, intramolecular hydrogen bonds between NH^…^O or N^…^HO play an active role. On the other hand, the chemically crosslinked CC-L-PEI/TA^H^ and CC-L-PEI/TA^C^ structures were crosslinked with GDE, due to the reaction of the epoxy ring with both -OH and -NH [8,32]. The prepared CC-L-PEI/TA hydrogels and cryogels can also be presumed to be copolymeric networks due to the crosslinking of L-PEI and TA molecules with each other [39,40]. The digital camera images of the PC-L-PEI/TA^H^-2, CC-L-PEI/TA^H^-2, and CC-L-PEI/TA^C^-2 networks were also given in Figure 3b–d. It was clearly seen that the color of networks darkens with increasing amounts of TA, confirming the presence of higher amounts of TA in the network.

The FT−IR spectra and TGA thermograms of L-PEI/TA-based networks were also compared in Figure 3e,f, respectively. When the FT−IR spectra of L-PEI/TA-based networks are examined in Figure 3e, the most prominent peaks observed that were different from the FT−IR spectra of L-PEI-based bare network structures were C−O vibration at 1200 cm^−1^ and C−OH stretching (ring) between 1370–1390 cm^−1^, respectively. Moreover, the comparison of the thermal stabilities of PC-L-PEI-TA^H^-2, CC-L-PEI/TA^H^-2, and CC-L-PEI/TA^C^-2 networks is given in Figure 3f. In a comparison of the thermal degradation profiles of PC-L-PEI-TA^H^-2, CC-L-PEI/TA^H^-2, and CC-L-PEI/TA^C^-2 networks, it was clearly seen that chemically crosslinked L-PEI/TA networks were thermally more stable than physically crosslinked forms. However, in comparison with the thermal stability of their TA-free forms, adding TA molecules to the structure slightly increased the thermal stability of L-PEI-based networks.

### 3.3. TA Release from L-PEI/TA-Based Hydrogels and Cryogels

**Figure 4:**
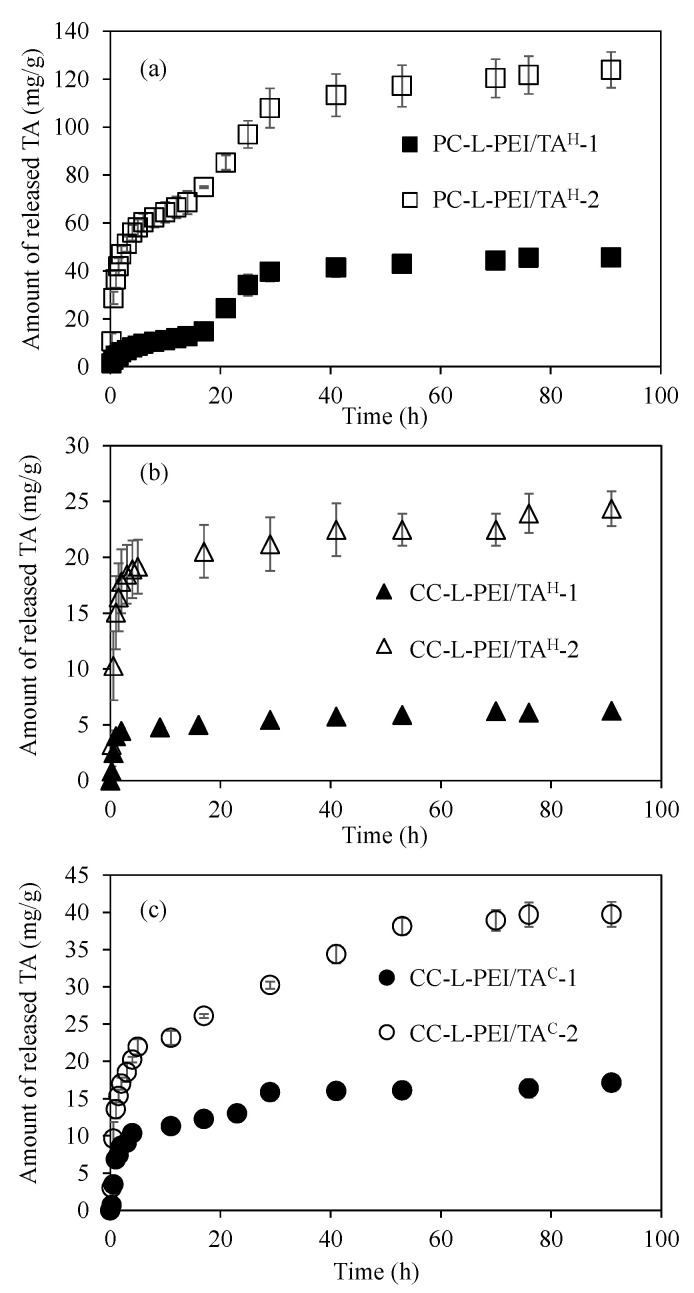
TA release profiles from (**a**) PC-L-PEI/TA^H^, (**b**) CC-L-PEI/TA^H^, and (**c**) CC-L-PEI/TA^C^ networks in pH 7.4 PBS at 37 °C.

The controllable release of TA can be very important because of the well-known radical scavenging, anti-inflammatory, neuro-protective, and antiviral effects, as well as its fairly good antimicrobial properties against various microorganisms such as yeast, fungi, or bacteria [19,20,22]. Therefore, the release study of TA from the prepared L-PEI/TA-based networks at pH 7.4 PBS at 37 °C was investigated, and corresponding graphs are given in Figure 4. The TA release from PC-L-PEI/TA^H^-1 and PC-L-PEI/TA^H^-2 at pH 7.4 PBS at 37 °C is presented in Figure 4a. The linear and rapid releasing of TA from both PC-L-PEI/TA^H^-1 and PC-L-PEI/TA^H^-2 networks was observed up to 6 h with 9.5 ± 0.5 mg/g and 60.2 ± 3.8 mg/g, respectively, which were equal to 10 and 25% of the TA content of PC-L-PEI/TA^H^-1 and PC-L-PEI/TA^H^-2. Interestingly, a second linear TA release was observed from both PC-L-PEI/TA^H^-1 and PC-L-PEI/TA^H^-2 networks between 17–29 h, with 39.6 ± 3.9 mg/g (40% of TA content) and 107.9 ± 8.2 mg/g (43% of TA content), respectively. Moreover, the TA release from PC-L-PEI/TA^H^-1 and PC-L-PEI/TA^H^-2 slightly continued up to 91 h with 45.6 ± 3.0 mg/g and 123.8 ± 7.5 mg/g cumulative releases of TA, which were almost 50% of the TA content of related networks, respectively.

On the other hand, a lesser amount of TA release was observed from CC-L-PEI/TA^H^-1 and CC-L-PEI/TA^H^-2 at pH 7.4 PBS at 37 °C, and the corresponding graph is given in Figure 4b. The linear release of TA from CC-L-PEI/TA^H^-1 and CC-L-PEI/TA^H^-2 networks was observed up to 2 h with a release amount of 4.4 ± 0.1 mg/g (4% of TA content) and 17.8 ± 3.0 mg/g (7% of TA content). Then, the release of TA from CC-L-PEI/TA^H^-1 and CC-L-PEI/TA^H^-2 networks slightly continued up to 91 h, with the release of almost 6% and 10% of TA content from relevant networks. Similarly, the TA release from CC-L-PEI/TA^C^-1 and CC-L-PEI/TA^C^-2 networks was also calculated and found to be less than the releasing amount of TA from physically crosslinked L-PEI/TA networks but also higher than the chemically crosslinked L-PEI/TA hydrogel networks. It can be clearly seen from Figure 4c that the linear releasing of TA from CC-L-PEI/TA^C^-1 and CC-L-PEI/TA^C^-2 networks was exhibited up to 4 and 5 h with 10.3 ± 0.4 mg/g (10% of TA content) and 21.9 ± 1.0 mg/g (9% of TA content), respectively. Also, another slow linear release was also observed between 4–29 h for CC-L-PEI/TA^C^-1 networks with 15.9 ± 1.1 mg/g cumulative release of TA, and 38.1 ± 1.1 mg/g cumulative TA release was observed from CC-L-PEI/TA^C^-2 networks after a slow linear release between 5–53 h.

In general, linear release was observed for all physically and chemically crosslinked L-PEI/TA hydrogels and cryogels; however, the observed linear release time showed variability depending on the crosslinking type. The physically crosslinked L-PEI/TA^H^ networks released more TA linearly than both the chemically crosslinked L-PEI/TA^H^ and L-PEI/TA^C^ networks. This can be explained by the ready degradation of hydrogen bonds between L-PEI and TA molecules, whereas chemical crosslinking via covalent bonds prevents the release of TA from CC-L-PEI/TA networks. On the other hand, it was also observed that the time for linear TA release and the released amounts of TA from CC-L-PEI/TA^C^ networks were higher than CC-L-PEI/TA^H^ networks. This can also be explained by the interconnected super-porous structure of cryogels.

### 3.4. Antioxidant Properties of L-PEI/TA-Based Hydrogels and Cryogels

The antioxidant activities of L-PEI/TA-based networks were investigated via total phenolic content (TPC) and ABTS^+^ scavenging assays. The concentrations of the supernatant solutions of L-PEI/TA-based networks taken in the first three hours of the TA release and the supernatant solutions of PC-L-PEI/TA^H^-1, PC-L-PEI/TA^H^-2, CC-L-PEI/TA^H^-1, CC-L-PEI/TA^H^-2, CC-L-PEI/TA^C^-1, and CC-L-PEI/TA^C^-2 were measured at 12.2, 48.5, 3.1, 12.3, 5.1 and 12.6 mg/mL, respectively. The results of the FC values and TEAC of the L-PEI/TA-based networks are given in Table 2.

As seen in Table 2, the concentration of TA solution prepared from the released TA solutions from the PC-L-PEI/TA^H^-1, PC-L-PEI/TA^H^-2, CC-L-PEI/TA^H^-1, CC-L-PEI/TA^H^-2, CC-L-PEI/TA^C^-1, and CC-L-PEI/TA^C^-2 networks directly affected the antioxidant properties of the system, as expected. The antioxidant equivalent capacities of PC-L-PEI/TA^H^-1, PC-L-PEI/TA^H^-2, CC-L-PEI/TA^H^-1, CC-L-PEI/TA^H^-2, CC-L-PEI/TA^C^-1 and CC-L-PEI/TA^C^-2 were calculated as 24.28 ± 2, 53.64 ± 5.3, 8.94 ±1.45, 25.46 ±4, 8.25 ± 1.9 and 23.07 ± 2.5 µg/mL gallic acid equivalent, respectively, while TEAC values were 0.29 ± 0.01, 0.48 ± 0.02, 0.05 ± 0.01, 0.26 ± 0.01, 0.04 ± 0.01 and 0.24 ± 0.03 µmol TE/g, respectively. Both TPC and TEAC test results affirmed that the L-PEI-based materials have prominent antioxidant properties that can be useful in various bio-medicinal applications, e.g., in the reduction of oxidative stress.

### 3.5. Antibacterial Properties of L-PEI and L-PEI/TA-Based Hydrogels and Cryogels

The antimicrobial activities of the prepared L-PEI and L-PEI/TA-based networks were investigated by means of a broth macro-dilution test and zone of inhibition experiments against Gram-negative bacteria, *E. coli*, and Gram-positive bacteria, *B. subtilis*. In the macro-dilution test, the lowest concentration values with no turbidity observed in tubes were determined as the minimum inhibitory concentration (MIC). Moreover, the lowest concentration values that decreased the number of organisms by >99.99% were determined as the minimum bactericidal concentration (MBC). MIC and MBC values of prepared L-PEI and L-PEI/TA-based networks are represented in Table 3.

**Figure 5:**
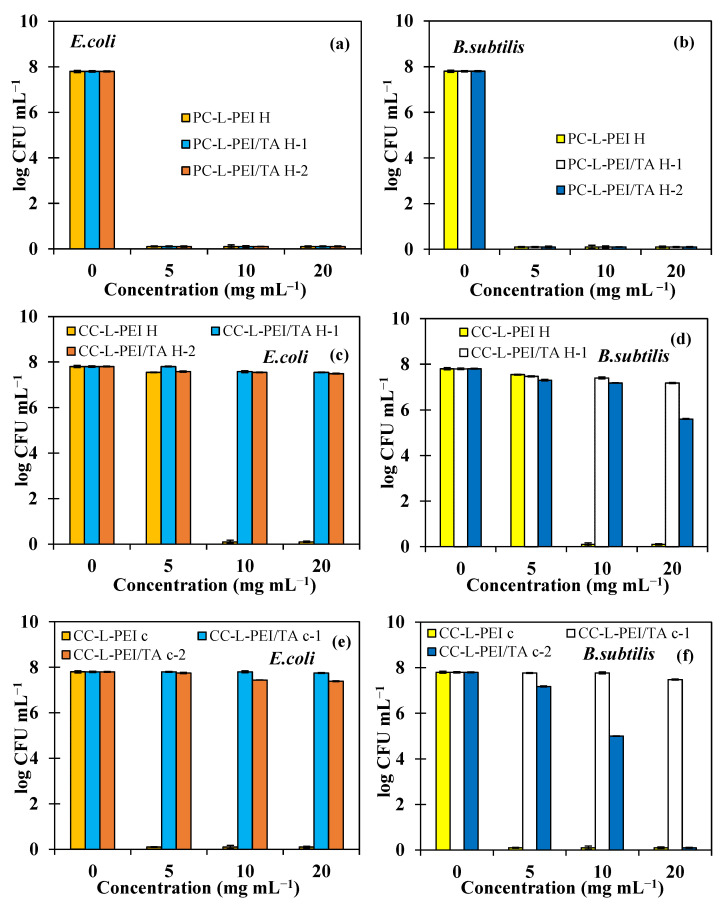
Antibacterial activities of PC-L-PEI H-based materials against (**a**) *E. coli* (**b**) *S. aureus*, CC-L-PEI H-based materials against (**c**) *E. coli* (**d**) *S. aureus*, and CC-L-PEI C-based materials against (**e**) *E. coli* (**f**) *S. aureus* are illustrated as bacterial growth numbers (log colony forming units (CFU)/mL).

As seen in Table 3, the MIC and MBC values of PC-L-PEI^H^, PC-L-PEI/TA^H^-1, and PC-L-PEI/TA^H^-2 were found to be 5 mg/mL against both Gram-negative and Gram-positive bacteria strains. The MIC values of CC-L-PEI^H^ were assessed at 5 mg/mL, and the MBC values were determined at 10 mg/mL against both *E. coli* and *B. subtilis.* The MIC values of CC-L-PEI/TA^H^-1 were found to be 20 mg/mL against *E. coli*, whereas the MIC and MBC values of the same material were determined to be 5 and 20 mg/mL, respectively, against *B. subtilis*. MIC values of CC-L-PEI/TA^H^-2 were 10 and 5 mg/mL against *E. coli* and *B. subtilis*, respectively. The MIC and MBC values of CC-L-PEI^C^ were 5 mg/mL against both bacteria strains. The MIC and MBC values of CC-L-PEI/TA^C^-1 were 20 mg/mL against B. subtilis, whereas CC-L-PEI/TA^C^-1 did not show any antibacterial effect against *E. coli* up to 20 mg/mL concentrations. MIC values of CC-L-PEI/TA^C^-1 against *E. coli* and *B. subtilis* were 10 and 5 mg/mL, respectively, whereas the MBC value was 20 mg/mL against *B. subtilis*. It is apparent that PC-L-PEI^H^-based materials and CC-L-PEI^C^ showed the highest antimicrobial activity of all prepared materials within 24 h of incubation. The higher antibacterial activity profile of prepared cryogels could be attributed to the much more rapid release of TA from the cryogel matrices. In general, it can be inferred that prepared L-PEI-based antimicrobial materials were more efficient against Gram-positive bacteria than Gram-negative bacteria. In the macro-dilution test, the inhibition of bacterial growth was also measured and illustrated in Figure 5.

In the macro-dilution test, the growth inhibitory effects of the prepared materials at 5, 10, and 20 mg/mL concentrations were evaluated within 24 h of incubation. Hence, the observed antibacterial effects were directly related to the released amount of TA from the materials. Materials with a rapid TA release profile were expected to exhibit higher antibacterial activity. The results are illustrated in Figure 5 as the number of bacteria colonies for Gram-negative *E. coli* and Gram-positive *B. subtilis* bacteria strains. As can be seen in Figure 5a,b, PC-L-PEI^H^, PC-L-PEI/TA^H^-1, and PC-L-PEI/TA^H^-2 samples, even at 5 mg/mL concentration, killed >99% of both bacteria strains and were determined as MBC. As shown in Figure 5c,d, CC-L-PEI^H^ at 5 mg/mL concentration killed 50% of both bacteria strains and was determined as the MIC value, whereas the same material at 10 mg/mL killed >99% of both strains and was determined as the MBC value. CC-L-PEI/TA^H^-1 at 20 mg/mL and 5 mg/mL, inhibited the growth of *E. coli* and *B. subtilis* strains, respectively, by 50% and were defined as MICs; the same material at 20 mg/mL caused toxicity on bacterial cells against *B. subtilis* and was calculated as the MBC. CC-L-PEI/TA^H^-2 at 10 and 5 mg/mL inhibited bacterial proliferation by 50% against *E. coli* and *B. subtilis*, respectively, as seen in Figure 5c. As shown in Figure 5d,e, CC-L-PEI^C^, even at a concentration of 5 mg/mL inhibited bacterial growth and reproduction 100% against both bacteria strains and was determined as the MBC value. Despite the fact that CC-L-PEI/TA^C^-1 did not show any significant antibacterial activity against *E. coli* up to 20 mg/mL, the same material at 20 mg/mL inhibited bacterial growth by 100% against *B. subtilis* as illustrated in Figure 5f. For *E. coli*, 280 CFU/mL formation at 10 mg/mL was detected in the medium containing CC-L-PEI/TA^C^-2, while 10^7^ CFU/mL of bacteria formed in the control group of CC-L-PEI/TA^C^-2 at 5 and 20 mg/mL inhibited the growth of *B. subtilis* by 50% and 100%, respectively. The experimental results of the macro-dilution test confirm the MIC and MBC values detected on nutrient broth-containing tubes.

On the other hand, zone of inhibition tests were performed to investigate the antibacterial effects of dry (lyophilized) hydrogel/cryogel composites, weighing 1.5 mg, on solid growth media at a 24 h incubation time, and zone diameters (mm) are given in Table 4.

**Figure 6:**
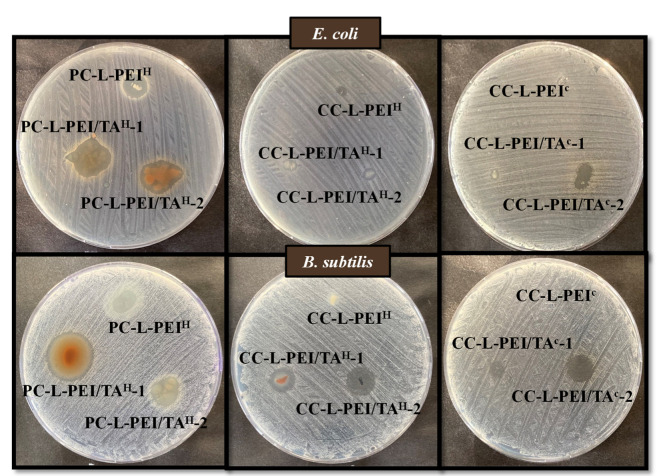
Zone of inhibition images of L-PEI-based hydrogel/cryogel samples weighing 1.5 mg against *E. coli* and *B. subtilis*.

As seen in Table 4, the zone diameters of PC-L-PEI^H^, PC-L-PEI/TA^H^-1, and PC-L-PEI/TA^H^-1 were 10 ± 0.5, 16 ± 0.5, and 14 ± 0.5 mm against *E. coli*, whereas they were 10 ± 1.5, 21 ± 0.5, and 12 ± 1.2 mm against *B. subtilis*, respectively. Zone diameters of CC-L-PEI^H^, CC-L-PEI/TA^H^-1, and CC-L-PEI/TA^H^-2 were 3 ± 0.5, 5 ± 0.5, and 4 ± 0.5 mm against *E. coli*, whereas they were 5 ± 0.5, 11 ± 1, and 12 ± 0.5 mm against *B. subtilis*, respectively. The zone diameters of CC-L-PEI^C^, CC-L-PEI/TA^C^-1, and CC-L-PEI/TA^C^-2 were 3 ± 0.5, 2 ± 0.5, and 7 ± 0.5 mm against *E. coli*, while they were 1 ± 0.5, 6 ± 1, and 11 ± 0.5 mm against *B. subtilis*, respectively. The results of the zone of inhibition experiments are also given in Figure 6, along with images of the zones that appeared on the nutrient agar medium.

It was determined that certain materials diffused to the nutrient agar surface at a higher rate than other materials after 24 h of incubation. As shown in Figure 6, CC-L-PEI/TA^C^-2 compared to CC-L-PEI/TA^C^-1 samples and PC-L-PEI/TA^H^-1 compared to PC-L-PEI/TA^H^-2 samples provided larger zone diameters. This is consistent, considering the faster tannic acid release profiles of PC-L-PEI/TA^H^-1 and PC-L-PEI/TA^H^-2. The spread of antimicrobial materials on the agar surface resulted in a larger zone diameter. In addition, similar to the results of the macro-dilution test, the prepared materials were more effective against the Gram-positive bacteria *B. subtilis* than Gram-negative bacteria *E. coli*. It can be said that even a relatively small amount of prepared L-PEI-based materials, e.g., 1.5 mg, can create significant inhibition zones, which is quite promising.

Today, nosocomial infections and antibiotic resistance have become serious problems. Resistance to many first-line antimicrobial agents has been reported, especially in the treatment of infections caused by the *E. coli* pathogen [41]. This highlights the need for agents that can be used as alternatives to currently used and frequently prescribed antibiotics. Prepared antimicrobial materials, especially PC-L-PEI^H^, PC-L-PEI/TA^H^-1, and PC-L-PEI/TA^H^-2, were highly effective against both Gram-negative and Gram-positive bacteria strains. Moreover, all the prepared materials gave promising results due to their rapid release and thus high antibacterial effect within 24 h. Therefore, prepared L-PEI and L-PEI/TA networks are candidates to be used in various applications in the biomedical field, such as in storage boxes of surgical instruments used by medical personnel or in environments where microorganisms can easily reproduce, such as denture containers.

## 4. Conclusions

The synthesis of L-PEI via the hydrolysis of PEOX in acidic conditions was successfully carried out. The disappearance of C = O amide vibrations at 1633 cm^−1^ and the appearance of N-H stretching vibrations at 3276 cm^−1^ confirmed the successful synthesis of L-PEI. After that, the prepared L-PEI was used to prepare physically and chemically crosslinked hydrogels and cryogels. The physically crosslinked PC-L-PEI^H^ networks were formed with hydrogen bonds between NH^…^N. On the other hand, GDE was used as a crosslinker for the preparation of chemically crosslinked CC-L-PEI^H^ and CC-L-PEI^C^ networks with epoxy-amine reactions. The gel yield % values for each L-PEI-based network were calculated at more than 95%. The higher S%, P%, and PV% values were observed for CC-L-PEI^C^ networks due to their interconnected, super porous structure. Moreover, the physically and chemically cross-linked L-PEI/TA hydrogels and cryogels containing 10% and 25% TA (w/w) with respect to L-PEI chains were prepared and shown to exhibit controllable TA release in pH 7.4 PBS at 37 °C. It was revealed that the physically crosslinked PC-L-PEI/TA^H^-1 and PC-L-PEI/TA^H^-2 networks resulted in linear TA release up to 6 h with 9.5 ± 0.5 mg/g, and 60.2 ± 3.8 mg/g, and also a second long-term release was observed between 17–29 h of release time with a release amount of 39.6 ± 3.9 mg/g (40% of TA content), and a 107.9 ± 8.2 mg/g (43% of TA content) TA, respectively. On the other hand, the release amounts of TA from chemically crosslinked CC-L-PEI/TA^H^ and CC-L-PEI/TA^C^ networks were found to be lesser in comparison to PC-L-PEI/TA^H^ networks, due to the crosslinking of both L-PEI and TA. The cross-linking of TA molecules to each other and to L-PEI chains prevented higher TA release from the structures. Moreover, the antioxidant activities of L-PEI/TA based networks were investigated via total phenolic content (TPC) and ABTS^+^ scavenging assays from released TA in pH 7.4 PBS at 37 °C in 3 h. It was observed that the higher release attained for the PC-L-PEI/TA^H^-2 network showed higher antioxidant activity as 53.64 ± 5.3 µg/mL gallic acid equivalent and 0.48 ± 0.02 µmol TA/g, respectively. On the other hand, depending on the released TA amount in pH 7.4 PBS at 37 °C in 3 h, all samples showed antioxidant activity. The MBC of prepared PC-L-PEI^H^ and CC-L-PEI^C^ networks against both Gram-negative *E. coli* and Gram-positive *B. subtilis* bacteria was determined at 5 mg/mL, whereas 10 mg/mL for CC-L-PEI^H^ networks against the same bacteria from the macro-dilution assay was attained. On the other hand, the calculated inhibition zone diameters for L-PEI and L-PEI/TA-based networks from the disc diffusion method showed that the increase in the amount of TA in L-PEI-based networks increased the zone diameters. The higher inhibition zone diameters against Gram-negative *E. coli*, and Gram-positive *B. subtilis* bacteria were determined to be 16 ± 0.5 and 21 ± 0.5 mm for PC-L-PEI/TA^H^-1 networks, respectively. The release of therapeutically effective concentrations of pharmaceuticals showed the viability of the physically and chemically crosslinked L-PEI-based networks as prospective drug carriers. Additionally, the use of various buffer solutions at various pH values for the release study can result in more sustained drug release. Owing to their antioxidant capabilities and controllable release profile, L-PEI-TA-based cryogels are promising materials to be used as plasters, dermal patches, or other scaffolds for covering certain wound types on the skin. The results of this study could be used for future in vivo experiments.

## Figures and Tables

**Table 1:** Swelling rate (S%), porosity (P%), pore volume (V_p_), and gel yield % of physically and chemically crosslinked L-PEI networks.

Materials	S%	P%	PV%	Gel Yield%
PC-L-PEI^H^	512 ± 54	51 ± 4	34 ± 3	95 ± 2%
CC-L-PEI^H^	976 ± 107	19 ± 2	7 ± 1	96 ± 1%
CC-L-PEI^C^	1154 ± 142	63 ± 5	57 ± 2	96 ± 1%

**Table 2:** Antioxidant activity of L-PEI-based materials, TPC value, and TEAC (determined as the antioxidant activity of the released TA from the materials within 3 h at 37 °C).

Samples	Concentration (mg/mL)	TPC (µg/mL)	TEAC (µmole TE/g)
PC-L-PEI/TA^H^-1	12.17	24.28 ± 2	0.29 ± 0.01
PC-L-PEI/TA^H^-2	48.51	53.64 ± 5.3	0.48 ± 0.02
CC-L-PEI/TA^H^-1	3.08	8.94 ± 1.45	0.05 ± 0.01
CC-L-PEI/TA^H^-2	12.32	25.46 ± 4	0.26 ± 0.01
CC-L-PEI/TA^C^-1	5.10	8.25 ± 1.9	0.04 ± 0.01
CC-L-PEI/TA^C^-2	12.57	23.07 ± 2.5	0.24 ± 0.03

**Table 3:** Minimum inhibition concentration (MIC) and minimum bactericidal concentration (MBC) values of L-PEI-based materials against *E. coli* and *B. subtilis* strains.

Bacteria	*E. coli*		*B. subtilis*	
Antimicrobial Materials	MIC (mg/mL)	MBC (mg/mL)	MIC (mg/mL)	MBC (mg/mL)
PC-L-PEI^H^	5	5	5	5
PC-L-PEI/TA^H^-1	5	5	5	5
PC-L-PEI/TA^H^-2	5	5	5	5
CC-L-PEI^H^	5	10	5	10
CC-L-PEI/TA^H^-1	20	ND	5	20
CC-L-PEI/TA^H^-2	10	ND	5	ND
CC-L-PEI^C^	5	5	5	5
CC-L-PEI/TA^C^-1	ND	ND	20	20
CC-L-PEI/TA^C^-2	10	ND	5	20

**Table 4:** Inhibition zone diameters (mm) of L-PEI-based materials against *E. coli* and *B. subtilis* strains.

Materials	Zone Diameter (mm)	
	*E. coli*	*B. subtilis*
PC-L-PEI^H^	10 ± 0.5	10 ± 1.5
PC-L-PEI/TA^H^-1	16 ± 0.5	21 ± 0.5
PC-L-PEI/TA^H^-2	14 ± 0.5	12 ± 1.2
CC-L-PEI^H^	3 ± 0.5	5 ± 0.5
CC-L-PEI/TA^H^-1	5 ± 0.5	11 ± 1
CC-L-PEI/TA^H^-2	4 ± 0.5	12 ± 0.5
CC-L-PEI^C^	3 ± 0.5	1 ± 0.5
CC-L-PEI/TA^C^-1	2 ± 0.5	6 ± 1
CC-L-PEI/TA^C^-2	7 ± 0.5	11 ± 0.5

## Data Availability

The data presented in this study are available on request from the corresponding author.

## References

[1] Ateia M., Arifuzzaman M., Pellizzeri S., Attia M.F., Tharayil N., Anker J.N., Karanfil T. (2019). Cationic polymer for selective removal of GenX and short-chain PFAS from surface waters and wastewaters at ng/L levels. Water Res..

[2] Zhou J., Xia K., Liu X., Fang L., Du H., Zhang X. (2021). Utilization of cationic polymer-modified fly ash for dye wastewater treatment. Clean Technol. Environ. Policy.

[3] Li X., Li Y., Wang H., Niu Z., He Y., Jin L., Wu M., Wang H., Chai L., Al-Enizi A.M. (2021). 3D Cationic Polymeric Network Nanotrap for Efficient Collection of Perrhenate Anion from Wastewater. Small.

[4] Chen Z., Lv Z., Sun Y., Chi Z., Qing G. (2020). Recent advancements in polyethyleneimine-based materials and their biomedical, biotechnology, and biomaterial applications. J. Mater. Chem. B.

[5] Dong R., Zhou L., Wu J., Tu C., Su Y., Zhu B., Gu H., Yan D., Zhu X. (2011). A supramolecular approach to the preparation of charge-tunable dendritic polycations for efficient gene delivery. Chem. Commun..

[6] Danko M., Kronekova Z., Krupa I., Tkac J., Matúš P., Kasak P. (2021). Exchange Counterion in Polycationic Hydrogels: Tunability of Hydrophobicity, Water State, and Floating Capability for a Floating pH Device. Gels.

[7] Sahiner N., Demirci S. (2017). Can PEI microgels become biocompatible upon betainization?. Mater. Sci. Eng. C.

[8] Bagdat S., Tokay F., Demirci S., Yilmaz S., Sahiner N. (2023). Removal of Cd(II), Co(II), Cr(III), Ni(II), Pb(II) and Zn(II) ions from wastewater using polyethyleneimine (PEI) cryogels. J. Environ. Manag..

[9] Lv Y., Yang S.-J., Du Y., Yang H.-C., Xu Z.-K. (2018). Co-deposition Kinetics of Polydopamine/Polyethyleneimine Coatings: Effects of Solution Composition and Substrate Surface. Langmuir.

[10] Ratanajanchai M., Soodvilai S., Pimpha N., Sunintaboon P. (2014). Polyethylenimine-immobilized core–shell nanoparticles: Synthesis, characterization, and biocompatibility test. Mater. Sci. Eng. C.

[11] Zou Y., Li D., Shen M., Shi X. (2019). Polyethylenimine-Based Nanogels for Biomedical Applications. Macromol. Biosci..

[12] Cai H., An X., Cui J., Li J., Wen S., Li K., Shen M., Zheng L., Zhang G., Shi X. (2013). Facile Hydrothermal Synthesis and Surface Functionalization of Polyethyleneimine-Coated Iron Oxide Nanoparticles for Biomedical Applications. ACS Appl. Mater. Interfaces.

[13] Ciofani G., Raffa V., Menciassi A., Cuschieri A. (2008). Cytocompatibility, interactions, and uptake of polyethyleneimine-coated boron nitride nanotubes by living cells: Confirmation of their potential for biomedical applications. Biotechnol. Bioeng..

[14] Kim H., Namgung R., Singha K., Oh I.-K., Kim W.J. (2011). Graphene Oxide–Polyethylenimine Nanoconstruct as a Gene Delivery Vector and Bioimaging Tool. Bioconjug. Chem..

[15] Hosu O., Florea A., Cristea C., Sandulescu R. (2019). Functionalized Advanced Hybrid Materials for Biosensing Applications. Advanced Biosensors for Health Care Applications.

[16] Huang Q., Liu M., Zhao J., Chen J., Zeng G., Huang H., Tian J., Wen Y., Zhang X., Wei Y. (2018). Facile preparation of polyethylenimine-tannins coated SiO2 hybrid materials for Cu2+ removal. Appl. Surf. Sci..

[17] Koopmann A.-K., Schuster C., Torres-Rodríguez J., Kain S., Pertl-Obermeyer H., Petutschnigg A., Hüsing N. (2020). Tannin-Based Hybrid Materials and Their Applications: A Review. Molecules.

[18] Guo Z., Xie W., Lu J., Guo X., Xu J., Xu W., Chi Y., Takuya N., Wu H., Zhao L. (2021). Tannic acid-based metal phenolic networks for bio-applications: A review. J. Mater. Chem. B.

[19] Kaczmarek B. (2020). Tannic Acid with Antiviral and Antibacterial Activity as A Promising Component of Biomaterials—A Minireview. Materials.

[20] Nakayama M., Suzuki K., Toda M., Okubo S., Hara Y., Shimamura T. (1993). Inhibition of the infectivity of influenza virus by tea polyphenols. Antivir. Res..

[21] Youness R., Kamel R., Elkasabgy N., Shao P., Farag M. (2021). Recent Advances in Tannic Acid (Gallotannin) Anticancer Activities and Drug Delivery Systems for Efficacy Improvement; A Comprehensive Review. Molecules.

[22] Chung K.-T., Wong T.Y., Wei C.-I., Huang Y.-W., Lin Y. (1998). Tannins and Human Health: A Review. Crit. Rev. Food Sci. Nutr..

[23] Fraga-Corral M., Otero P., Cassani L., Echave J., Garcia-Oliveira P., Carpena M., Chamorro F., Lourenço-Lopes C., Prieto M.A., Simal-Gandara J. (2021). Traditional Applications of Tannin Rich Extracts Supported by Scientific Data: Chemical Composition, Bioavailability and Bioaccessibility. Foods.

[24] Hatami E., Nagesh P., Chowdhury P., Chauhan S., Jaggi M., Samarasinghe A., Yallapu M. (2018). Tannic Acid-Lung Fluid Assemblies Promote Interaction and Delivery of Drugs to Lung Cancer Cells. Pharmaceutics.

[25] Huang Y., Wu D., Bao M., Li B., Liang H. (2019). Coordination driven self-assembly for enhancing the biological stability of nobiletin. J. Mol. Liq..

[26] Chowdhury P., Nagesh P.K.B., Hatami E., Wagh S., Dan N., Tripathi M.K., Khan S., Hafeez B.B., Meibohm B., Chauhan S.C. (2019). Tannic acid-inspired paclitaxel nanoparticles for enhanced anticancer effects in breast cancer cells. J. Colloid Interface Sci..

[27] Liang X., Cao K., Li W., Li X., McClements D.J., Hu K. (2021). Tannic acid-fortified zein-pectin nanoparticles: Stability, properties, antioxidant activity, and in vitro digestion. Food Res. Int..

[28] Leite L.S.F., Pham C., Bilatto S., Azeredo H.M.C., Cranston E.D., Moreira F.K., Mattoso L.H.C., Bras J. (2021). Effect of Tannic Acid and Cellulose Nanocrystals on Antioxidant and Antimicrobial Properties of Gelatin Films. ACS Sustain. Chem. Eng..

[29] Soradech S., Williams A.C., Khutoryanskiy V.V. (2022). Physically Cross-Linked Cryogels of Linear Polyethyleneimine: Influence of Cooling Temperature and Solvent Composition. Macromolecules.

[30] Kowalczyk A., Ruszkiewicz M., Biskup I. (2015). Total phenolic content and antioxidant capacity of polish apple ciders. Indian J. Pharm. Sci..

[31] Ivanauskas R., Bronusiene A., Ivanauskas A., Šarkinas A., Ancutiene I. (2022). Antibacterial Activity of Copper Particles Embedded in Knitted Fabrics. Materials.

[32] Ari B., Sahiner M., Demirci S., Sahiner N. (2022). Poly(Vinyl alcohol)-tannic Acid Cryogel Matrix as Antioxidant and Antibacterial Material. Polymers.

[33] Reisner B.S., Woods G.L. (1999). Times to Detection of Bacteria and Yeasts in BACTEC 9240 Blood Culture Bottles. J. Clin. Microbiol..

[34] Reller L.B. (1973). Endocarditis Caused by Bacillus subtilis. Am. J. Clin. Pathol..

[35] Sedlacek O., Janouskova O., Verbraeken B., Hoogenboom R. (2019). Straightforward Route to Superhydrophilic Poly(2-oxazoline)s via Acylation of Well-Defined Polyethylenimine. Biomacromolecules.

[36] Shan X., Williams A.C., Khutoryanskiy V.V. (2020). Polymer structure and property effects on solid dispersions with haloperidol: Poly(N-vinyl pyrrolidone) and poly(2-oxazolines) studies. Int. J. Pharm..

[37] Lozinsky V.I. (2008). Polymeric cryogels as a new family of macroporous and supermacroporous materials for biotechnological purposes. Russ. Chem. Bull..

[38] Lozinsky V.I., Galaev I.Y., Plieva F.M., Savina I.N., Jungvid H., Mattiasson B. (2003). Polymeric cryogels as promising materials of biotechnological interest. Trends Biotechnol..

[39] Feng X., Fan J., Li A., Li G. (2020). Biobased Tannic Acid Cross-Linked Epoxy Thermosets with Hierarchical Molecular Structure and Tunable Properties: Damping, Shape Memory, and Recyclability. ACS Sustain. Chem. Eng..

[40] Zhou S., Yan J., Chen J., Yan H., Zhang Y., Huang J., Zhao G., Zhang Q., Liu Y. (2023). Polydopamine/polyethyleneimine co-crosslinked graphene oxide for the enhanced tribological performance of epoxy resin coatings. J. Mater. Sci. Technol..

[41] Nimer N.A. (2022). Nosocomial Infection and Antibiotic-Resistant Threat in the Middle East. Infect. Drug Resist..

